# *catena*-Poly[[aqua­bromidocopper(II)]-μ_3_-(picolinato *N*-oxide)]

**DOI:** 10.1107/S1600536811001814

**Published:** 2011-01-22

**Authors:** Xin-Yu Wang, Xiao-Qing Zhang, Wen-Shi Wu

**Affiliations:** a College of Materials Science and Engineering, Huaqiao University, Xiamen, Fujian 361021, People’s Republic of China

## Abstract

The title complex, [CuBr(C_6_H_4_NO_3_)(H_2_O)]_*n*_, exhibits a layered structure which is stabilized by inter­molecular O—H⋯O and O—H⋯Br^−^ hydrogen bonds, van der Waals forces and π–π inter­actions [centroid–centroid distance = 3.747(4) Å] between the parallel pyridine rings from two neighboring layers.

## Related literature

For the isotypic chlorido complex, see: Yang *et al.* (2004 ▶). For the synthesis, see: Wu *et al.* (2007 ▶). 
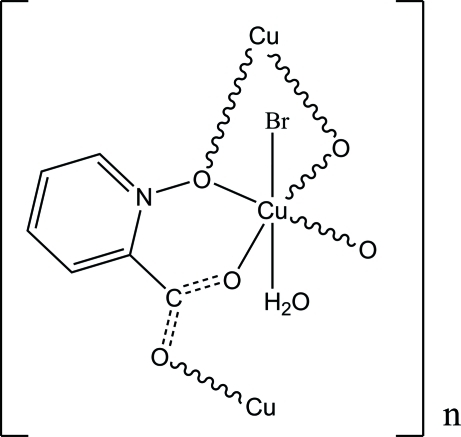


## Experimental

### 

#### Crystal data

[CuBr(C_6_H_4_NO_3_)(H_2_O)]
*M* *_r_* = 299.57
Monoclinic, 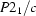

*a* = 9.7116 (3) Å
*b* = 10.0302 (2) Å
*c* = 9.4984 (3) Å
β = 110.821 (2)°
*V* = 864.81 (4) Å^3^
*Z* = 4
Mo *K*α radiation
μ = 7.12 mm^−1^
*T* = 173 K
0.52 × 0.35 × 0.22 mm

#### Data collection

Bruker SMART CCD area-detector diffractometer
Absorption correction: multi-scan (*SADABS*; Sheldrick, 1996 ▶) *T*_min_ = 0.095, *T*_max_ = 0.241
2584 measured reflections
1515 independent reflections
1420 reflections with *I* > 2σ(*I*)
*R*_int_ = 0.027

#### Refinement

*R*[*F* ^2^ > 2σ(*F* ^2^)] = 0.044
*wR*(*F* ^2^) = 0.127
*S* = 1.00
1515 reflections
118 parameters
H-atom parameters constrained
Δρ_max_ = 1.09 e Å^−3^
Δρ_min_ = −0.93 e Å^−3^

### 

Data collection: *SMART* (Bruker, 1999 ▶); cell refinement: *SAINT* (Bruker, 1999 ▶); data reduction: *SAINT*; program(s) used to solve structure: *SHELXS97* (Sheldrick, 2008 ▶); program(s) used to refine structure: *SHELXL97* (Sheldrick, 2008 ▶); molecular graphics: *SHELXTL* (Sheldrick, 2008 ▶); software used to prepare material for publication: *SHELXTL*.

## Supplementary Material

Crystal structure: contains datablocks global, I. DOI: 10.1107/S1600536811001814/hg2780sup1.cif

Structure factors: contains datablocks I. DOI: 10.1107/S1600536811001814/hg2780Isup2.hkl

Additional supplementary materials:  crystallographic information; 3D view; checkCIF report

## Figures and Tables

**Table 1:** Hydrogen-bond geometry (Å, °)

*D*—H⋯*A*	*D*—H	H⋯*A*	*D*⋯*A*	*D*—H⋯*A*
O4—H4*B*2⋯O2^i^	0.85	1.97	2.738 (5)	149
O4—H4*B*2⋯Br1^i^	0.85	3.07	3.741 (4)	137
O4—H4*B*1⋯Br1^ii^	0.85	2.59	3.377 (4)	155

Symmetry codes: (i) 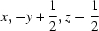; (ii) 
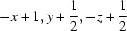
.

**Fig. 1.**
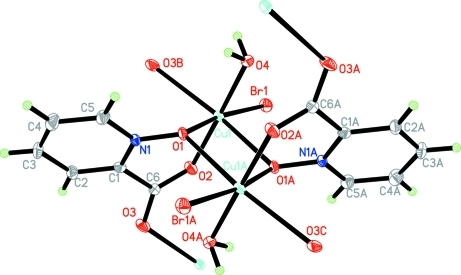
ORTEP drawing (at 30% probability) of the compound structure. [Symmetry code: (A) 1-x, 1-y, 1-z (B) x, 0.5-y, -0.5+z (C) 1-x, 0.5+y, 1.5-z].

**Fig. 2.**
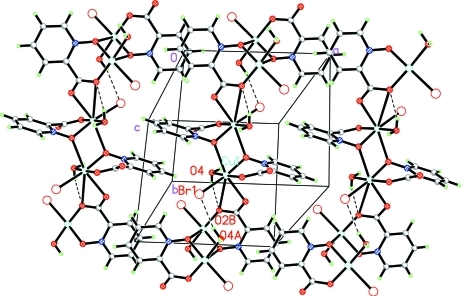
Crystal Packing diagram of the title compound, showing the H-bonded interactions (dashed lines),π-π interactions diagram. [Symmetry code: (A) 1-x, -0.5+y, 0.5-z (B) x, 0.5-y, -0.5+z].

**Fig. 3.**
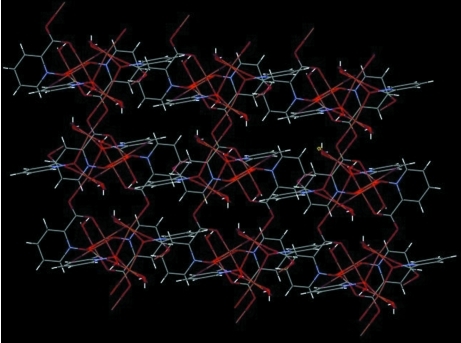
Packing diagram.

**Fig. 4.**
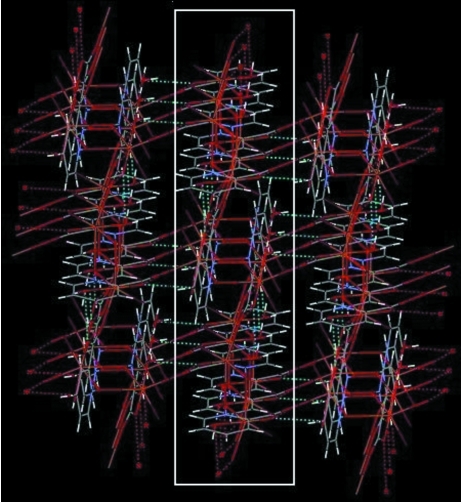
Packing diagram.

## supplementary crystallographic information

### Comment

The title complex, [(C_6_H_4_NO_3_)(H_2_O)BrCu]_n_, is isomorphous with the chloro complex (Yang *et al.*, 2004). The atoms of C1,C2,C3,C4,C5,C6,N1 and O1 lie in a plane with r.m.s. of 0.0095Å (Figure 1). The Cu(II) ion is 6-coordinated by a bidentate picolinate N-oxide chelating ligand (O1 and O2), a N-oxide oxygen atom and a carboxylate oxygen atom from two other ligands, an aqua ligand and a bromine anion (Figure 2). The two axial Cu—O bond lengths Cu-O1 and Cu-O3 are 2.449 (4) and 2.499 (4)Å and are longer than those reported for the chloride complex (Yang *et al.*, 2004), while the Cu—O4 bond length of 1.990 (4)Å is shorter. The distance of Cu—Br is 2.403 (8)Å.

The complex exhibits a layered crystal structure which is stabilized by intermolecular O—H···O and O—H···Br^-^ hydrogen bonds, van der Waals forces and π-π interactions between  parallel pyridine rings from two neighboring layers (Figure 2). The distances between the layers are 3.318 (2) Å. The title complex forms Cu_2_O_2_ units interconnected *via* 2-carboxylic acid-pyridine-N-oxide ligands, and such unit formed a parallelogram (Figure 2).

### Experimental

The title complex was synthesized according to the method of Wu *et al.*, (2007). The CuBr_2_ (0.1 g, 0.5 mmol) was dissolved in 20 ml methanol(20 ml), then 2-carboxylic acid-pyridine-N-oxide (0.07 g, 0.5 mmol) in THF (20 ml) was added slowly. The mixture was then stirred for a few hours. Brown crystals of the title complex were grown from the mother liquor by slow evapovation after three weeks.

### Refinement

The position of the water H atoms were located in a difference Fourier map. However, during refinement, they were restrained to O—H=0.85 Å. The *U*_iso_ of each H atom = 1.5*U*_eq_(O). The C-bound H atoms were included in the riding model approximation with C—H = 0.95 Å. The *U*_iso_ of each H atom = 1.2*U*_eq_(C).

### Crystal data

**Table:**

[CuBr(C_6_H_4_NO_3_)(H_2_O)]	*F*(000) = 580
*M**_r_* = 299.57	*D*_x_ = 2.301 Mg m^−^^3^
Monoclinic, *P*2_1_/*c*	Mo *K*α radiation, λ = 0.71073 Å
Hall symbol: -P 2ybc	Cell parameters from 1539 reflections
*a* = 9.7116 (3) Å	θ = 2.2–25.1°
*b* = 10.0302 (2) Å	µ = 7.12 mm^−^^1^
*c* = 9.4984 (3) Å	*T* = 173 K
β = 110.821 (2)°	Prism, brown
*V* = 864.81 (4) Å^3^	0.52 × 0.35 × 0.22 mm
*Z* = 4	

### Data collection

**Table:**

Bruker SMART CCD area-detector diffractometer	1515 independent reflections
Radiation source: fine-focus sealed tube	1420 reflections with *I* > 2σ(*I*)
graphite	*R*_int_ = 0.027
Detector resolution: 0 pixels mm^-1^	θ_max_ = 25.1°, θ_min_ = 2.2°
φ and ω scans	*h* = −8→11
Absorption correction: multi-scan (*SADABS*; Sheldrick, 1996)	*k* = −11→11
*T*_min_ = 0.095, *T*_max_ = 0.241	*l* = −10→11
2584 measured reflections	

### Refinement

**Table:**

Refinement on *F*^2^	Primary atom site location: structure-invariant direct methods
Least-squares matrix: full	Secondary atom site location: difference Fourier map
*R*[*F*^2^ > 2σ(*F*^2^)] = 0.044	Hydrogen site location: inferred from neighbouring sites
*wR*(*F*^2^) = 0.127	H-atom parameters constrained
*S* = 1.00	*w* = 1/[σ^2^(*F*_o_^2^) + (0.081*P*)^2^ + 5.867*P*] where *P* = (*F*_o_^2^ + 2*F*_c_^2^)/3
1515 reflections	(Δ/σ)_max_ = 0.012
118 parameters	Δρ_max_ = 1.09 e Å^−^^3^
0 restraints	Δρ_min_ = −0.93 e Å^−^^3^

### Special details

**Table:**

Geometry. All e.s.d.'s (except the e.s.d. in the dihedral angle between two l.s. planes) are estimated using the full covariance matrix. The cell e.s.d.'s are taken into account individually in the estimation of e.s.d.'s in distances, angles and torsion angles; correlations between e.s.d.'s in cell parameters are only used when they are defined by crystal symmetry. An approximate (isotropic) treatment of cell e.s.d.'s is used for estimating e.s.d.'s involving l.s. planes.
Refinement. Refinement of *F*^2^ against ALL reflections. The weighted *R*-factor *wR* and goodness of fit *S* are based on *F*^2^, conventional *R*-factors *R* are based on *F*, with *F* set to zero for negative *F*^2^. The threshold expression of *F*^2^ > σ(*F*^2^) is used only for calculating *R*-factors(gt) *etc*. and is not relevant to the choice of reflections for refinement. *R*-factors based on *F*^2^ are statistically about twice as large as those based on *F*, and *R*- factors based on ALL data will be even larger.

### Fractional atomic coordinates and isotropic or equivalent isotropic displacement parameters (Å^2^)

**Table:**

	*x*	*y*	*z*	*U*_iso_*/*U*_eq_	
Cu1	0.49099 (7)	0.34456 (6)	0.41130 (7)	0.0175 (2)	
N1	0.2180 (5)	0.4606 (4)	0.3842 (5)	0.0152 (9)	
O1	0.3577 (4)	0.4929 (4)	0.4067 (4)	0.0184 (8)	
O2	0.4245 (4)	0.2618 (4)	0.5602 (4)	0.0256 (9)	
O3	0.2807 (5)	0.2489 (5)	0.6967 (5)	0.0294 (10)	
C1	0.1845 (6)	0.3627 (6)	0.4668 (6)	0.0193 (12)	
C2	0.0387 (7)	0.3350 (6)	0.4398 (7)	0.0238 (13)	
H2A	0.0140	0.2691	0.4987	0.029*	
C3	−0.0713 (6)	0.4001 (7)	0.3300 (7)	0.0315 (15)	
H3A	−0.1715	0.3771	0.3092	0.038*	
C4	−0.0343 (7)	0.5010 (7)	0.2490 (8)	0.0338 (15)	
H4A	−0.1092	0.5496	0.1744	0.041*	
C5	0.1116 (7)	0.5293 (6)	0.2782 (7)	0.0259 (13)	
H5A	0.1377	0.5978	0.2232	0.031*	
C6	0.3066 (6)	0.2862 (5)	0.5851 (6)	0.0180 (11)	
Br1	0.67758 (6)	0.17677 (6)	0.45034 (7)	0.0260 (2)	
O4	0.5513 (4)	0.4396 (4)	0.2586 (4)	0.0216 (8)	
H4B2	0.5328	0.3904	0.1812	0.032*	
H4B1	0.4763	0.4764	0.1945	0.032*	

### Atomic displacement parameters (Å^2^)

**Table:**

	*U*^11^	*U*^22^	*U*^33^	*U*^12^	*U*^13^	*U*^23^
Cu1	0.0175 (4)	0.0176 (4)	0.0202 (4)	0.0022 (2)	0.0101 (3)	0.0019 (2)
N1	0.013 (2)	0.015 (2)	0.018 (2)	0.0020 (16)	0.0062 (18)	0.0016 (17)
O1	0.0139 (19)	0.0198 (19)	0.023 (2)	−0.0029 (14)	0.0085 (16)	−0.0027 (15)
O2	0.023 (2)	0.030 (2)	0.028 (2)	0.0079 (18)	0.0140 (19)	0.0088 (18)
O3	0.031 (2)	0.037 (2)	0.027 (2)	0.0078 (19)	0.0185 (19)	0.0154 (19)
C1	0.023 (3)	0.018 (3)	0.018 (3)	−0.001 (2)	0.009 (2)	−0.002 (2)
C2	0.019 (3)	0.025 (3)	0.028 (3)	−0.003 (2)	0.009 (3)	−0.002 (2)
C3	0.015 (3)	0.042 (4)	0.034 (4)	−0.005 (3)	0.005 (3)	−0.004 (3)
C4	0.024 (3)	0.039 (4)	0.033 (3)	0.008 (3)	0.003 (3)	0.007 (3)
C5	0.023 (3)	0.028 (3)	0.024 (3)	0.004 (2)	0.005 (2)	0.007 (2)
C6	0.019 (3)	0.016 (3)	0.019 (3)	0.000 (2)	0.006 (2)	0.001 (2)
Br1	0.0240 (4)	0.0251 (4)	0.0299 (4)	0.0071 (2)	0.0110 (3)	0.0003 (2)
O4	0.021 (2)	0.025 (2)	0.018 (2)	−0.0014 (16)	0.0058 (16)	−0.0016 (16)

### Geometric parameters (Å, °)

**Table:**

Cu1—O2	1.938 (4)	C1—C6	1.520 (8)
Cu1—O1	1.963 (4)	C2—C3	1.365 (9)
Cu1—O4	1.990 (4)	C2—H2A	0.9500
Cu1—Br1	2.4034 (8)	C3—C4	1.393 (10)
N1—O1	1.336 (6)	C3—H3A	0.9500
N1—C5	1.348 (7)	C4—C5	1.373 (9)
N1—C1	1.366 (7)	C4—H4A	0.9500
O2—C6	1.272 (7)	C5—H5A	0.9500
O3—C6	1.232 (7)	O4—H4B2	0.8500
C1—C2	1.375 (8)	O4—H4B1	0.8500
			
O2—Cu1—O1	87.31 (16)	C1—C2—H2A	119.3
O2—Cu1—O4	176.34 (17)	C2—C3—C4	119.0 (6)
O1—Cu1—O4	89.04 (16)	C2—C3—H3A	120.5
O2—Cu1—Br1	90.90 (12)	C4—C3—H3A	120.5
O1—Cu1—Br1	171.71 (12)	C5—C4—C3	119.3 (6)
O4—Cu1—Br1	92.68 (12)	C5—C4—H4A	120.4
O1—N1—C5	117.5 (4)	C3—C4—H4A	120.4
O1—N1—C1	121.2 (4)	N1—C5—C4	120.5 (6)
C5—N1—C1	121.4 (5)	N1—C5—H5A	119.8
N1—O1—Cu1	116.3 (3)	C4—C5—H5A	119.8
C6—O2—Cu1	127.5 (4)	O3—C6—O2	125.1 (5)
N1—C1—C2	118.5 (5)	O3—C6—C1	116.4 (5)
N1—C1—C6	120.3 (5)	O2—C6—C1	118.5 (5)
C2—C1—C6	121.1 (5)	Cu1—O4—H4B2	109.1
C3—C2—C1	121.3 (6)	Cu1—O4—H4B1	109.3
C3—C2—H2A	119.3	H4B2—O4—H4B1	76.6
			
C5—N1—O1—Cu1	−131.2 (4)	C1—C2—C3—C4	−3.0 (10)
C1—N1—O1—Cu1	49.5 (5)	C2—C3—C4—C5	2.1 (10)
O2—Cu1—O1—N1	−52.2 (3)	O1—N1—C5—C4	179.8 (5)
O4—Cu1—O1—N1	128.0 (3)	C1—N1—C5—C4	−0.9 (9)
O1—Cu1—O2—C6	20.4 (5)	C3—C4—C5—N1	−0.1 (10)
Br1—Cu1—O2—C6	−167.7 (5)	Cu1—O2—C6—O3	−166.6 (4)
O1—N1—C1—C2	179.2 (5)	Cu1—O2—C6—C1	15.8 (7)
C5—N1—C1—C2	0.0 (8)	N1—C1—C6—O3	147.3 (5)
O1—N1—C1—C6	−1.1 (7)	C2—C1—C6—O3	−33.0 (8)
C5—N1—C1—C6	179.6 (5)	N1—C1—C6—O2	−34.8 (8)
N1—C1—C2—C3	2.0 (9)	C2—C1—C6—O2	144.8 (6)
C6—C1—C2—C3	−177.7 (6)		

### Hydrogen-bond geometry (Å, °)

**Table:**

*D*—H···*A*	*D*—H	H···*A*	*D*···*A*	*D*—H···*A*
O4—H4B2···O2^i^	0.85	1.97	2.738 (5)	149
O4—H4B2···Br1^i^	0.85	3.07	3.741 (4)	137
O4—H4B1···Br1^ii^	0.85	2.59	3.377 (4)	155

Symmetry codes: (i) *x*, −*y*+1/2, *z*−1/2; (ii) −*x*+1, *y*+1/2, −*z*+1/2.
